# Urinary parabens and breast cancer risk: Modification by LINE‐1 and LUMA global DNA methylation, and associations with breast cancer defined by tumor promoter methylation status

**DOI:** 10.1002/mc.23456

**Published:** 2022-08-17

**Authors:** Humberto Parada, Leili Sahrai, Mary S. Wolff, Regina M. Santella, Jia Chen, Alfred I. Neugut, Susan L. Teitelbaum

**Affiliations:** ^1^ School of Public Health, Division of Epidemiology and Biostatistics San Diego State University San Diego California USA; ^2^ UC San Diego Moores Cancer Center La Jolla California USA; ^3^ Department of Environmental Medicine and Public Health Icahn School of Medicine at Mount Sinai New York New York USA; ^4^ Department of Environmental Health Sciences, Mailman School of Public Health Columbia University New York New York USA; ^5^ Department of Medicine and Department of Epidemiology, Mailman School of Public Health, Vagelos College of Physicians and Surgeons Columbia University New York New York USA

**Keywords:** breast cancer, DNA methylation, endocrine disrupting chemicals, gene promoter methylation, parabens

## Abstract

Parabens are a group of alkyl esters of *p*‐hydroxybenzoic acid added to consumer products to prevent the growth of harmful bacteria and molds. Parabens are hypothesized to increase the risk of breast cancer (BC); however, no study has examined the interactions between parabens, global DNA methylation (DNAm), and BC risk. We examined the modifying effects of DNAm on the associations between parabens and BC, and whether parabens were associated with BC defined by tumor promoter methylation status. Participants included 708 cases and 598 controls from the Long Island Breast Cancer Study Project. Methylparaben (MPB), propylparaben, and butylparaben levels were measured in spot urine samples. Global DNAm was measured by analysis of long interspersed elementes‐1 (LINE‐1) and the luminometric methylation assay (LUMA). The promoter methylation status of 13 genes was measured in tumor samples from 509 cases. We used logistic regression to estimate odds ratios (ORs) and 95% confidence intervals (CIs) for the associations between parabens and BC stratified by LINE‐1/LUMA, and between parabens and gene‐specific promoter methylation‐defined BC. Outcome heterogeneity was evaluated using ratios of ORs (RORs). We assessed the joint effects of the multiple parabens using quantile g‐computation. The highest versus lowest tertile of MPB and a one‐quantile increase in all parabens were associated with ORs of 1.46 (95% CI = 0.96–2.23) and 1.32 (95% CI = 1.02–1.71), respectively, among women with hypomethylated LINE‐1. A one‐ln unit increase in MPB was associated with a 25% increase in the odds of hypomethylated (vs. hypermethylated) *CCND2* promoter‐defined BC (ROR = 1.25, 95% CI = 1.06–1.48), and a one‐quantile increase in all parabens was associated with a 55% increase in the odds of hypomethylated (vs. hypermethylated) *CCND2* promoter‐defined BC (ROR = 1.55, 95% CI = 1.04–2.32). Exposure to parabens may increase the risk of BC among women with hypomethylated global DNAm and may increase the risk of tumors with gene‐specific hypomethylated promoter regions.

## 1 INTRODUCTION

Personal care and consumer products, such as cosmetics and pharmaceuticals, are an important source of exposure to parabens, a group of alkyl esters of *p*‐hydroxybenzoic acid.^1^ The parabens most commonly added and the most frequently detected in humans include methylparaben (MPB) and propylparaben (PPB), and to a lesser extent butylparaben (BPB).^2^ Parabens are often used in combination or with other chemicals as preservatives to prevent the growth of harmful bacteria and molds.^3^ Many paraben‐containing products are used habitually and are applied and left on the skin, which permits parabens to be absorbed into the bloodstream.^4^ Once absorbed, parabens are hydrolyzed by esterase in the skin, small intestine, and liver before being excreted in the urine^5^; however, it is unclear whether the relatively lower levels of esterase in the skin^6^ are sufficient to hydrolyze all dermally absorbed parabens. Parabens that enter the body intact can bind to human serum albumin protecting them from hydrolysis^5^ and facilitating their detection in adipose tissues.^7^ Indeed, intact parabens can be measured in breast tissues^8^, ^9^ and in breast milk.^10^, ^11^

Owing to their potential to act as estrogens or interfere with the function of the estrogen receptor or its downstream pathways, parabens are hypothesized to increase the risk of breast cancer (BC),^12^ the most frequently diagnosed nonskin cancer and the second leading cause of cancer‐related death among US women.^13^ Despite their ubiquitous exposure and potential as mammary carcinogens,^12^ to date only two epidemiologic studies^14^, ^15^ have examined parabens in association with BC. In the Long Island Breast Cancer Study Project (LIBCSP), we reported increases in the odds of BC among women with the highest versus the lowest quintiles of urinary MPB, PPB, and the sum of MPB, PPB, and BPB.^14^ A recent study by Wu et al., however, reported no increases in BC in a nested case‐control study with estimates below one comparing the highest versus lowest tertiles of ethylparaben, MPB, and PPB, and a statistically significant inverse association reported comparing the highest versus lowest tertiles of total parabens.^15^

Expanding on our previous work, herein we examined whether the associations between parabens and BC^14^ were modified by global DNA methylation status measured in peripheral blood DNA using two independent assays of global methylation markers, pyrosequencing of long interspersed elementes‐1 (LINE‐1) and the luminometric methylation assay (LUMA).^16^ The LINE‐1‐based assay selectively measures the methylation of CpG islands located in long interspersed elements whereas LUMA measures the methylation of overall 5‐methylcytosine content in “CCGG” recognition sites.^17^ Thus, while both assays quantify global DNA methylation, they are thought to measure different DNA methylation facets of epigenetic regulation of gene expression given that they address different recognition sites within the genome.^18^ Global DNA hypomethylation and regional hypermethylation of specific genes are hallmarks of BC and may contribute to malignancy by activating oncogenes, silencing tumor suppressors and metastasis inhibitor genes, and inducing genomic instability.^19^ Environmental carcinogens, including parabens and global DNA methylation, may thus have synergistic effects on breast carcinogenesis. We also examined the associations between parabens and BCs defined by the tumor promoter methylation status of 13 genes with established roles in breast carcinogenesis frequently methylated in breast tumor tissues, and which are associated with the malignant BC phenotype.^20^ These included the promoters of steroid hormone genes (*ESR1*, *PGR*, and *RARβ*), tumor suppressor genes (*APC, BRCA1*, *CDH1*, *DAPK1, HIN1*, *P16*, and *RASSF1a*), an oncogene (*CCND2*), a detoxification gene (*GSTP1*), and a transcription factor gene (*TWIST1*).^21^, ^22^, ^23^, ^24^, ^25^, ^26^, ^27^, ^28^, ^29^, ^30^ Understanding whether parabens are associated with tumor promoter methylation‐defined BCs may shed light on the biological mechanisms by which parabens may increase BC risk. Last, given the potential additive, synergistic, or antagonistic effects of parabens, we also examined the joint impacts of the multiple paraben exposures using quantile g‐computation.^31^

## 2 METHODS

### 2.1 Study population and study design

We used resources from the LIBCSP, a population‐based case‐control study of BC.^32^ LIBCSP participants were adult residents of Nassau and Suffolk Counties on Long Island, NY. Cases were women with primary in situ or invasive BC diagnosed between August 1, 1996 and July 31, 1997 ascertained by rapid case ascertainment. Controls were women without BC who were frequency matched by age to cases in 5‐year groups. Controls were identified via random digit dialing for women under 65 and from Health Care Finance Administration rosters for women 65 years or older. LIBCSP participants were interviewed at home by trained interviewers using structured questionnaires. Cases were interviewed on average within 3 months of diagnosis. The majority of the participants also provided blood (93% of cases and 83% of controls) and urine (73% of cases and 73% of controls) samples at the time of the interview and most cases provided samples before receiving chemotherapy (77% for blood and 75% for urine). This study included 708 of the 1508 cases and 598 of the 1556 controls who had available data on urinary parabens^14^ and global DNA methylation.^16^

The LIBCSP protocol was approved by the Institutional Review Board of all participating institutions and written informed consent was obtained from participants before data collection. The analysis of blinded specimens by the Centers for Disease Control and Prevention (CDC) laboratory was determined not to constitute any engagement in human subjects research.

### 2.2 Quantification of urinary parabens

Details regarding the processing and storage of urine samples and laboratory procedures have been previously described.^14^, ^33^ In brief, urine samples were analyzed at the National Center for Environmental Health for MPB, BPB, and PPB in 2007 and 2010 using online solid‐phase extraction followed by high‐performance liquid chromatography‐isotope dilution tandem mass spectrometry. Detection frequencies were >90% for MPB and PPB and 50% for BPB. Values below the limit of detection (LOD) were imputed as the LOD divided by the square root of 2, and urinary levels (μg/L) were divided by creatinine for final units of micrograms per gram (μg/g) of creatinine. For MPB and PPB, levels were categorized into tertiles based on the distributions in the controls. Given the high proportion of samples with nondetectable levels of BBP, we used quantiles that categorized values with nondetectable concentrations into the lowest exposure group and values with detectable levels dichotomized at the median among the controls.

### 2.3 Global DNA methylation

Global DNA methylation was measured in DNA extracted from peripheral blood samples from cases and controls. The methylation status of four CpG sites in the promoter region of LINE‐1 was measured using a pyrosequencing‐based methylation assay as previously described.^16^ In brief, genomic DNA was treated with bisulfite using Zymo DNA methylation kits (Zymo Research), and eluted in elution buffer before polymerase chain reaction (PCR). PCR products were sequenced using the Pyrosequencing PSQ96 HS System (Qiagen) and the methylation status at each of the four loci was analyzed as a T/C single‐nucleotide polymorphism using QCpG software (Qiagen). Methylation status data at all four loci were averaged to provide an overall percentage 5 mC status.

The methylation status of the C^m^CGG motif was measured using LUMA.^16^ In brief, genomic DNA was digested by *Hpa*II + *Eco*RI or *Msp*I + *Eco*RI in two separate reactions. To the digestion product, annealing buffer was added, and samples were analyzed with a PyroMark Q24 system (Qiagen) with the following dispensation order: GTGTCA‐ CATGTG. LUMA methylation level was expressed as a percentage as LUMA Methylation %=[1−(*Hpa*II ΣG/ΣT)/(*Msp*I ΣG/ΣT)]×100.^34^

For statistical analyses and to maximize power for stratified analyses, the continuous levels of LINE‐1/LUMA were dichotomized (≤median = hypomethylated versus >median = hypermethylated) based on the median methylation levels in controls.

### 2.4 Gene‐specific promoter DNA methylation

The promoter methylation status of 13 genes (*APC*, *BRCA1, CDH1, CCND2, DAPK1, ESR1, GSTP1, HIN1, PGR, P16*, *RARβ*, *RASSF1A*, and *TWIST1*) was measured in DNA from archived formalin‐fixed paraffin‐embedded tumor tissue samples from cases.^20^ Methylation of *ER*, *PR*, and *BRCA1* was determined by MSP‐PCR and was dichotomized (hypermethylated vs. hypomethylated) based on the presence or absence of the PCR band.^35^ The methylation status of the 10 remaining genes was assessed by the MethylLight assay. The percentage of methylation was calculated by the 2^−ΔΔC^_T_ method, using the following equation: ΔΔCT = (C_T,Target_ – C_T,Actin_) sample −(C_T,Target_ − C_T,Actin_) full methylated DNA × 100.^36^ For statistical analyses, we used a 4% cut‐off^37^ to dichotomize methylation of each gene (≤4% = hypomethylated and >4% = hypermethylated). Only 18 women had a hypermethylated *P16* promoter; we therefore excluded this gene from the analysis.

### 2.5 Covariates

Confounders of the association between paraben exposure and BC risk included: demographics (age [continuous] and education [<high school/high school graduate, college, or postcollege]); reproductive factors (menopausal status [premenopausal or postmenopausal], age at menarche [≤12 or >12 years], and parity and lactation history [nulliparous, parous/never lactated, or parous/ever lactated]); medical‐related factors (family history of BC [none or a first degree relative]); exogenous hormone use [hormone replacement therapy use (ever or never)]; and lifestyle/behavioral factors (body mass index in the year before diagnosis [≤25.0 or >25.0 kg/m^2^] and lifetime alcohol intake [nondrinker, <15, 15–29, ≥30g/day]).

### 2.6 Statistical analysis

For single paraben analyses, we used multivariable logistic regression stratified by LINE‐1 or LUMA methylation status (dichotomized at the median in the controls) to estimate odds ratios (ORs) and 95% confidence intervals (95% CIs) for the associations between each paraben and incident BC. In‐text, we also provide CI limit ratios (CLRs; the upper limit divided by the lower limit), a measure of the CI precision.^38^ We evaluated effect measure modification on the multiplicative scale by including statistical interactions between ln‐transformed continuous paraben levels and global DNA methylation levels. For the multiple parabens analyses, we used quantile g‐computation^31^ in conjunction with logistic regression [family = binomial()], in fully adjusted models using the R package qgComp15 (version 2.3.0) to examine the associations between the multiple parabens simultaneously and odds of incident BC. For quantile g‐computation, the estimated coefficient ψ is interpretable as the log(OR) of increasing all exposures by one quantile at the same time. For the multiple paraben analyses, we evaluated effect measure modification on the multiplicative scale using the Cochrane *Q*‐test for heterogeneity.

For single paraben analyses, we used multinomial logistic regression to estimate ORs and 95% CIs for the associations between each paraben and incident BC defined by gene‐specific promoter methylation status (hypomethylated cases [≤4%] or hypermethylated cases [>4%] vs. controls). For the multiple parabens analyses, we also used quantile g‐computation in conjunction with logistic regression, in fully adjusted models to examine the associations between the multiple parabens simultaneously and incident BC defined by gene‐specific promoter methylation status. We evaluated outcome heterogeneity using ratios of the odds ratio (RORs) from logistic models regressing case status (hypomethylated vs. hypermethylated cases) on continuous ln‐transformed paraben levels and covariates.

Analyses were based on participants with complete data (i.e., complete‐case analysis) and were conducted using SAS Version 9.4 (SAS Institute Inc.) and R Studio (Version 1.4.1103; R Foundation for Statistical Computing).

## 3 RESULTS

The characteristics of the 708 cases and 598 controls included in this study are reported in Table 1. Compared to controls, cases were more likely to have less than a high school education (47.8% vs. 40.2%) and were in the postmenopausal stage (67.4% vs. 62.9%), overweight or obese (55.6% vs. 47.8%). Controls were more likely to have been parous and reported ever lactating (37.8%) compared to cases (34.2%). Based on the distributions of LINE‐1 and LUMA in the controls, higher proportions of cases were above than below the medians for LINE‐1 (52.0%) and LUMA (63.5%).

**Table 1:** Distribution of select characteristics among the LIBSCP women with data on urinary parabens and LINE‐1/LUMA DNA methylation by case‐control status (*n*= 1306)

Characteristic	*n* (%)	
	Cases	Controls
*n*	708 (100)	598 (100)
Age (years)		
<50	195 (27.5)	197 (32.9)
50–64	258 (36.4)	250 (41.8)
≥65	255 (36.0)	151 (25.3)
Education		
<HS/HS graduate	338 (47.8)	240 (40.2)
College	261 (36.9)	263 (44.1)
Postcollege	108 (15.3)	94 (15.7)
Missing	1	1
Menopausal status		
Premenopausal	225 (32.6)	211 (37.1)
Postmenopausal	466 (67.4)	357 (62.9)
Missing	17	30
HRT use		
Never	504 (71.3)	439 (73.4)
Ever	203 (28.7)	159 (26.6)
Missing	1	0
Age at menarche (years)		
≤12	309 (44.0)	274 (46.1)
>12	393 (56.0)	321 (53.9)
Missing	6	3
Parity/lactation history		
Nulliparous	92 (13.0)	70 (11.7)
Parous/never lactated	374 (52.8)	302 (50.5)
Parous/ever lactated	242 (34.2)	226 (37.8)
Family history of breast cancer		
None	561 (82.0)	498 (84.7)
First degree	123 (18.0)	90 (15.3)
Missing	24	10
BMI at reference (kg/m^2^)		
<25.0	314 (44.5)	309 (52.2)
25.0–29.9	220 (31.2)	160 (27.0)
≥30	172 (24.4)	123 (20.8)
Missing	2	6
Lifetime alcohol intake (g/day)		
Nondrinkers	263 (37.1)	225 (37.7)
<15	336 (47.5)	289 (48.4)
15–30	71 (10.0)	48 (8.0)
≥30	38 (5.4)	35 (5.9)
Missing	0	1
LINE‐1		
≤78.6%	338 (48.0)	296 (50.0)
>78.6%	366 (52.0)	296 (50.0)
Missing		6
LUMA		
≤56.3%	256 (36.5)	297 (50.1)
>56.3%	445 (63.5)	296 (49.9)
Missing	7	5

*Note*: LIBCSP population‐based women without breast cancer were frequency matched by age to women diagnosed with breast cancer between August 1, 1996 and July 31, 1997.

Abbreviations: BMI, body mass index; HRT, hormone replacement therapy; HS, high school; LIBCSP, Long Island Breast Cancer Study Project; LINE‐1, long interspersed elementes‐1; LUMA, luminometric methylation assay.

### 3.1 Effect modification by global DNA methylation

Higher versus lower levels of MPB, BPB, and the multiple parabens were associated with increased odds of BC and more so among women with hypomethylated than hypermethylated LINE‐1, as reported in Table 2. Among women in the hypomethylated LINE‐1 stratum, there was a 46% increase in odds of BC (OR = 1.46, 95% CI = 0.96–2.23; CLR = 2.32) among women in the highest (vs. lowest) tertile of MPB, which was attenuated among women in the hypermethylated LINE‐1 stratum (OR = 1.12, 95% CI = 0.74–1.69; CLR = 2.28); CI, however, included the null value. Increasing all parabens by one tertile/quantile at the same time (quantile g‐computation weights were 0.494 for MPB, 0.268 for PPB, and 0.238 for BPB) was associated with a 32% increase in the odds of BC (OR = 1.32, 95% CI = 1.02–1.71; ψ = 0.279, SE = 0.133; CLR = 1.68) among women in the hypomethylated LINE‐1 stratum, but not among women in the hypermethylated LINE‐1 stratum. However, the interactions were not statistically significant (*P*_Interation_≥0.10).

**Table 2:** Multivariable logistic regression odds ratios (ORs) and 95% confidence intervals (CIs) for the associations between creatinine‐corrected urinary paraben levels and breast cancer stratified by LINE‐1 and LUMA methylation status (*n*= 1306)

Paraben (μg/g creatinine)	Hypomethylated LINE‐1 (≤78.6%)			Hypermethylated LINE‐1 (>78.6%)			
	Ca/Co	OR^a^	95% CI^a^	Ca/Co	OR^a^	95% CI^a^	*p*_Interaction_ b
Methylparaben							
1.04–73.3	89/93	1.00	Reference	133/104	1.00	Reference	
73.4–267	124/100	1.50	0.98–2.29	113/96	0.99	0.66–1.50	
270–3174	125/103	1.46	0.96–2.23	120/96	1.12	0.74–1.69	
Ln(methylparaben)		1.13	1.00–1.27		1.06	0.94–1.18	0.53
Propylparaben							
<LOD–12.4	96/92	1.00	Reference	109/105	1.00	Reference	
12.5–67.7	117/106	1.27	0.84–1.94	133/91	1.72	1.15–2.59	
67.8–3116	125/98	1.40	0.92–2.14	124/100	1.44	0.95–2.18	
Ln(propylparaben)		1.05	0.96–1.15		1.07	0.98–1.17	0.10
Butylparaben							
<LOD	160/144	1.00	Reference	206/139	1.00	Reference	
LOD–2.49	79/75	1.18	0.78–1.80	79/80	0.71	0.47–1.06	
2.50–173	99/77	1.31	0.88–1.96	81/77	0.74	0.49–1.12	
Ln(butylparaben)		1.03	0.94–1.12		0.93	0.85–1.03	0.55
Multiple parabens^c^		1.32	1.02–1.71		0.98	0.76–1.27	0.11

**Paraben (μg/g creatinine)**	**Hypomethylated LUMA (≤56.3%)**			**Hypermethylated LUMA (>56.3%)**			
	**Ca/Co**	**OR** a	**95% CI** a	**Ca/Co**	**OR** a	**95% CI** a	*p* **_Interaction_** b
Methylparaben							
1.04–73.3	73/107	1.00	Reference	147/91	1.00	Reference	
73.4–267	94/93	1.57	1.00–2.46	145/103	0.98	0.66–1.45	
270–3174	89/97	1.41	0.90–2.21	153/102	1.13	0.76–1.68	
Ln(methylparaben)		1.11	0.98–1.27		1.06	0.95–1.18	0.58
Propylparaben							
<LOD–12.4	72/106	1.00	Reference	132/92	1.00	Reference	
12.5–67.7	97/95	1.70	1.09–2.66	152/101	1.24	0.84–1.85	
67.8–3116	87/96	1.50	0.95–2.36	161/103	1.26	0.84–1.88	
Ln(propylparaben)		1.07	0.97–1.18		1.03	0.95–1.13	0.09
Butylparaben							
<LOD	133/140	1.00	Reference	231/144	1.00	Reference	
LOD–2.49	59/73	0.99	0.63–1.55	101/82	0.87	0.59–1.28	
2.50–173	64/84	0.85	0.54–1.33	113/70	1.08	0.73–1.59	
Ln(butylparaben)		0.95	0.86–1.05		0.99	0.91–1.08	0.44
Multiple parabens^c^		1.16	0.88–1.52		1.09	0.86–1.40	0.71

*Note*: LIBCSP population‐based women without breast cancer were frequency matched by age to women diagnosed with breast cancer between August 1, 1996 and July 31, 1997.

Abbreviations: Ca, cases; CI, confidence interval; Co, controls; LIBCSP, Long Island Breast Cancer Study Project; LINE‐1, long interspersed elementes‐1; LOD, limit of detection; LUMA, luminometric methylation assay; OR, odds ratio.

^a^ Adjusted for age (continuous) and education (<high school/high school graduate, college, postcollege), menopausal status (premenopausal, post‐menopausal), age at menarche (≤12, >12 years), parity and lactation history (nulliparous, parous/never lactated, parous/ever lactated), family history of breast cancer (none or at least one first degree relative), hormone replacement therapy use (ever, never), body mass index in the year before diagnosis (≤25.0, >25.0 kg/m^2^), and lifetime alcohol intake (nondrinkers, <15, 15–29, ≥30g/day).

^b^ For single paraben analysis, *^p^*_Interaction_ is the *p* value from the multiplicative interaction from logistic regression models using continuous creatinine‐corrected paraben levels and global DNA methylation levels. For multiple paraben analysis, *^p^*_Interaction_ is the *p* value from the Cochrane *Q*‐test for heterogeneity.

^c^ Multiple paraben analysis estimated using quantile g‐computation.

Among women in the hypomethylated LUMA stratum, there were increased odds of BC among women in the highest (vs. lowest) tertiles of MPB (OR = 1.41, 95% CI = 0.90–2.21; CLR = 2.46) and PPB (OR = 1.50, 95% CI = 0.95–2.36; CLR = 2.48), which were attenuated among women in the hypermethylated LUMA stratum. These associations, however, were not statistically significant.

### 3.2 Heterogeneity of tumor promoter methylation‐defined BC

We observed statistically significant outcome heterogeneity for one (*CCND2)* of the 12 genes examined, which was apparent for MPB and BPB and for the multiple parabens, as reported in Table 3. For MPB, the highest (vs. lowest) tertile was associated with a nonstatistically significant increase in the odds of hypomethylated *CCND2* promoter‐defined BC (OR = 1.31, 95% CI = 0.93–1.84; CLR = 1.98) versus controls, and with a nonstatistically significant decrease in the odds of hypermethylated *CCND2* promoter‐defined BC (OR = 0.71, 95% CI = 0.40–1.25; CLR = 3.13). A one‐ln unit increase in MPB was associated with an OR of 1.11 (95% CI = 1.01–1.22; CLR = 1.21) for hypomethylated *CCND2* promoter‐defined BC and with an OR of 0.89 (95% CI = 0.76–1.03; CLR = 1.36) for hypermethylated *CCND2* promoter‐defined BC. There was a 25% increase in the odds of hypomethylated (vs. hypermethylated) *CCND2* promoter‐defined BC (ROR = 1.25, 95% CI = 1.06–1.48; CLR = 1.40) for a one ln‐unit increase in MPB.

**Table 3:** Multinomial logistic regression odds ratios (ORs) and 95% confidence intervals (CIs) for the associations between creatinine‐corrected urinary paraben levels and breast tumor gene promoter methylation status (*n*= 1113)

Gene promoter paraben (μg/g creatinine)	Breast tumor gene promoter methylation status						
	Hypomethylated cases vs. controls			Hypermethylated cases vs. controls			
	Ca/Co	OR^a^	95% CI	Ca/Co	OR^a^	95% CI	ROR^b^ (95% CI)
*APC*							
Methylparaben							
1.04–73.3	77/199	1.00	Reference	86/199	1.00	Reference	
73.4–267	93/199	1.37	0.93–2.02	72/199	0.96	0.65–1.42	
270–3174	76/200	1.08	0.72–1.63	81/200	1.11	0.75–1.63	
Ln(methylparaben)		1.05	0.94–1.17		1.04	0.94–1.16	1.00 (0.88–1.14)
Propylparaben							
<LOD–12.4	79/199	1.00	Reference	72/199	1.00	Reference	
12.5–67.7	84/199	1.29	0.87–1.90	81/199	1.34	0.90–1.99	
67.8–3116	83/200	1.21	0.81–1.80	86/200	1.46	0.98–2.18	
Ln(propylparaben)		1.02	0.94–1.11		1.07	0.98–1.16	0.96 (0.86–1.06)
Butylparaben							
<LOD	124/288	1.00	Reference	136/288	1.00	Reference	
LOD–2.49	61/155	1.05	0.71–1.54	54/155	0.86	0.58–1.27	
2.50–173	61/155	0.96	0.65–1.42	49/155	0.72	0.48–1.07	
Ln(butylparaben)		0.97	0.89–1.06		0.89	0.82–0.98	1.09 (0.98–1.21)
Multiple parabens^c^		1.07	0.84–1.37		1.00	0.78–1.28	1.07 (0.80–1.45)
*BRCA1*							
Methylparaben							
1.04–73.3	69/199	1.00	Reference	100/199	1.00	Reference	
73.4–267	70/199	1.20	0.79–1.81	103/199	1.15	0.80–1.65	
270–3174	68/200	1.21	0.79–1.84	103/200	1.15	0.80–1.65	
Ln(methylparaben)		1.05	0.94–1.18		1.07	0.97–1.19	0.98 (0.86–1.11)
Propylparaben							
<LOD–12.4	64/199	1.00	Reference	92/199	1.00	Reference	
12.5–67.7	72/199	1.39	0.91–2.11	103/199	1.37	0.95–1.98	
67.8–3,116	71/200	1.42	0.93–2.18	111/200	1.44	1.00–2.09	
Ln(propylparaben)		1.08	0.98–1.18		1.05	0.97–1.13	1.02 (0.92–1.13)
Butylparaben							
<LOD	112/288	1.00	Reference	162/288	1.00	Reference	
LOD–2.49	46/155	0.94	0.62–1.44	73/155	0.94	0.66–1.35	
2.50–173	49/155	0.94	0.62–1.42	71/155	0.83	0.58–1.20	
Ln(butylparaben)		0.95	0.87–1.05		0.95	0.87–1.03	1.00 (0.91–1.11)
Multiple parabens^c^		1.13	0.87–1.46		1.05	0.84–1.32	1.02 (0.76–1.36)
*CDH1*							
Methylparaben							
1.04–73.3	149/199	1.00	Reference	7/199	1.00	Reference	
73.4–267	145/199	1.12	0.81–1.54	8/199	0.97	0.33–2.87	
270–3174	145/200	1.13	0.82–1.57	11/200	1.42	0.51–3.98	
Ln(methylparaben)		1.06	0.97–1.16		1.03	0.78–1.37	1.04 (0.78–1.38)
Propylparaben							
<LOD–12.4	132/199	1.00	Reference	10/199	1.00	Reference	
12.5–67.7	151/199	1.37	0.99–1.90	7/199	0.68	0.23–1.99	
67.8–3116	156/200	1.41	1.02–1.97	9/200	1.05	0.39–2.81	
Ln(propylparaben)		1.06	0.99–1.13		0.93	0.75–1.16	1.12 (0.90–1.39)
Butylparaben							
<LOD	231/288	1.00	Reference	14/288	1.00	Reference	
LOD–2.49	107/155	0.99	0.72–1.36	5/155	0.88	0.30–2.61	
2.50–173	101/155	0.85	0.61–1.18	7/155	1.13	0.42–3.03	
Ln(butylparaben)		0.94	0.87–1.01		0.95	0.75–1.20	0.99 (0.78–1.25)
Multiple parabens^c^		1.06	0.87–1.30		1.01	0.53–1.93	1.02 (0.53–1.98)
*CCND2*							
Methylparaben							
1.04–73.3	118/199	1.00	Reference	38/199	1.00	Reference	
73.4–267	126/199	1.26	0.90–1.78	27/199	0.65	0.36–1.16	
270–3174	129/200	1.31	0.93–1.84	27/200	0.71	0.40–1.25	
Ln(methylparaben)		1.11	1.01–1.22		0.89	0.76–1.03	1.25 (1.06–1.48)
Propylparaben							
<LOD‐12.4	108/199	1.00	Reference	34/199	1.00	Reference	
12.5–67.7	130/199	1.41	1.00‐1.99	28/199	1.00	0.56‐1.80	
67.8–3116	135/200	1.48	1.04‐2.09	30/200	1.11	0.62‐1.97	
Ln(propylparaben)		1.07	1.00‐1.16		0.97	0.86‐1.09	1.10 (0.97–1.25)
<LOD	186/288	1.00	Reference	59/288	1.00	Reference	
LOD–2.49	92/155	1.05	0.75–1.46	20/155	0.76	0.42–1.35	
2.50–173	95/155	0.98	0.70–1.37	13/155	0.48	0.25–0.93	
Ln(butylparaben)		0.97	0.90–1.05		0.79	0.68–0.92	1.22 (1.05–1.41)
Multiple parabens^c^		1.16	0.94–1.43		0.74	0.50–1.08	1.55 (1.04–2.32)
*DAPK1*							
Methylparaben							
1.04–73.3	137/199	1.00	Reference	19/199	1.00	Reference	
73.4–267	124/199	1.05	0.75–1.46	29/199	1.57	0.82–3.01	
270–3174	135/200	1.16	0.83–1.62	21/200	1.12	0.55–2.24	
Ln(methylparaben)		1.07	0.97–1.17		1.03	0.86–1.22	1.05 (0.87–1.26)
Propylparaben							
<LOD–12.4	119/199	1.00	Reference	23/199	1.00	Reference	
12.5–67.7	134/199	1.32	0.94–1.84	24/199	1.32	0.68–2.54	
67.8–3116	143/200	1.43	1.02–2.00	22/200	1.22	0.62–2.39	
Ln(propylparaben)		1.06	0.99–1.14		1.00	0.87–1.15	1.06 (0.92–1.22)
Butylparaben							
<LOD	211/288	1.00	Reference	34/288	1.00	Reference	
LOD–2.49	96/155	0.97	0.70–1.35	16/155	1.02	0.52–1.99	
2.50–173	89/155	0.82	0.58–1.15	19/155	1.17	0.62–2.21	
Ln(butylparaben)		0.94	0.87–1.01		0.96	0.83–1.10	0.98 (0.84–1.13)
Multiple parabens^c^		1.06	0.86–1.31		1.07	0.71–1.60	0.93 (0.60–1.42)
*ESR1*							
Methylparaben							
1.04–73.3	99/199	1.00	Reference	68/199	1.00	Reference	
73.4–267	84/199	1.01	0.69–1.47	88/199	1.41	0.95–2.10	
270–3174	101/200	1.23	0.85–1.78	69/200	1.12	0.74–1.69	
Ln(methylparaben)		1.10	0.99–1.22		1.03	0.92–1.15	1.05 (0.93–1.20)
Propylparaben							
<LOD–12.4	81/199	1.00	Reference	74/199	1.00	Reference	
12.5–67.7	98/199	1.49	1.02–2.18	75/199	1.24	0.83–1.85	
67.8–3116	105/200	1.63	1.11–2.40	76/200	1.23	0.82–1.85	
Ln(propylparaben)		1.08	1.00–1.17		1.04	0.95–1.14	1.03 (0.93–1.14)
Butylparaben							
<LOD	140/288	1.00	Reference	131/288	1.00	Reference	
LOD–2.49	75/155	1.18	0.82–1.70	43/155	0.71	0.47–1.07	
2.50–173	69/155	1.01	0.70–1.47	51/155	0.76	0.51–1.14	
Ln(butylparaben)		0.98	0.90–1.06		0.93	0.85–1.02	1.04 (0.94–1.16)
Multiple parabens^c^		1.21	0.96–1.53		0.95	0.74–1.23	1.26 (0.94–1.68)
*GSTP1*							
Methylparaben							
1.04–73.3	112/199	1.00	Reference	44/199	1.00	Reference	
73.4–267	111/199	1.16	0.82–1.64	42/199	0.98	0.59–1.62	
270–3174	113/200	1.17	0.82–1.66	43/200	1.11	0.67–1.83	
Ln(methylparaben)		1.06	0.96–1.17		1.06	0.93–1.22	0.99 (0.86–1.15)
Propylparaben							
<LOD–12.4	100/199	1.00	Reference	42/199	1.00	Reference	
12.5–67.7	118/199	1.41	0.99–2.01	40/199	1.10	0.66–1.84	
67.8–3116	118/200	1.39	0.97–1.99	47/200	1.38	0.83–2.28	
Ln(propylparaben)		1.06	0.98–1.14		1.04	0.93–1.15	1.02 (0.91–1.14)
Butylparaben							
<LOD	180/288	1.00	Reference	65/288	1.00	Reference	
LOD–2.49	81/155	0.95	0.68–1.35	31/155	1.06	0.64–1.74	
2.50–173	75/155	0.80	0.56–1.14	33/155	1.05	0.64–1.73	
Ln(butylparaben)		0.94	0.87–1.01		0.95	0.85–1.06	0.97 (0.86–1.09)
Multiple parabens^c^		1.05	0.85–1.31		1.11	0.81–1.51	0.91 (0.64–1.28)
*HIN1*							
Methylparaben							
1.04–73.3	61/199	1.00	Reference	95/199	1.00	Reference	
73.4–267	43/199	0.75	0.47–1.20	110/199	1.34	0.94–1.92	
270–3174	64/200	1.18	0.76–1.82	92/200	1.13	0.77–1.64	
Ln(methylparaben)		1.06	0.93–1.20		1.06	0.96–1.17	1.00 (0.87–1.15)
Propylparaben							
<LOD–12.4	52/199	1.00	Reference	90/199	1.00	Reference	
12.5–67.7	52/199	1.11	0.70–1.76	106/199	1.45	1.00–2.09	
67.8–3116	64/200	1.42	0.90–2.23	101/200	1.37	0.94–1.99	
Ln(propylparaben)		1.06	0.96–1.17		1.04	0.96–1.13	1.02 (0.91–1.13)
Butylparaben							
<LOD	91/288	1.00	Reference	154/288	1.00	Reference	
LOD–2.49	38/155	0.91	0.58–1.42	74/155	1.02	0.71–1.46	
2.50–173	39/155	0.83	0.53–1.31	69/155	0.88	0.61–1.27	
Ln(butylparaben)		0.97	0.88–1.07		0.92	0.85–1.00	1.06 (0.95–1.18)
Multiple parabens^c^		1.06	0.80–1.40		1.08	0.86–1.36	1.00 (0.73–1.38)
*PGR*							
Methylparaben							
1.04–73.3	146/199	1.00	Reference	23/199	1.00	Reference	
73.4–267	159/199	1.27	0.92–1.75	14/199	0.61	0.28–1.29	
270–3174	147/200	1.18	0.85–1.63	24/200	1.12	0.58–2.17	
Ln(methylparaben)		1.06	0.97–1.16		1.08	0.89–1.32	1.00 (0.82–1.23)
Propylparaben							
<LOD–12.4	135/199	1.00	Reference	21/199	1.00	Reference	
12.5–67.7	158/199	1.46	1.05–2.02	17/199	0.88	0.43–1.83	
67.8–3116	159/200	1.47	1.06–2.05	23/200	1.21	0.61–2.39	
Ln(propylparaben)		1.07	0.99–1.14		1.02	0.88–1.19	1.05 (0.90–1.23)
Butylparaben							
<LOD	241/288	1.00	Reference	33/288	1.00	Reference	
LOD–2.49	102/155	0.91	0.66–1.26	17/155	1.17	0.61–2.24	
2.50–173	109/155	0.91	0.66–1.25	11/155	0.63	0.29–1.36	
Ln(butylparaben)		0.96	0.90–1.04		0.84	0.71–1.00	1.16 (0.97–1.38)
Multiple parabens^c^		1.10	0.90–1.35		0.94	0.60–1.47	1.22 (0.77–1.94)
*RARβ*							
Methylparaben							
1.04–73.3	109/199	1.00	Reference	47/199	1.00	Reference	
73.4–267	121/199	1.27	0.90–1.80	32/199	0.75	0.44–1.26	
270–3174	112/200	1.18	0.83–1.69	44/200	1.09	0.66–1.78	
Ln(methylparaben)		1.08	0.98–1.19		1.01	0.88–1.16	1.06 (0.91–1.22)
Propylparaben							
<LOD–12.4	98/199	1.00	Reference	44/199	1.00	Reference	
12.5–67.7	123/199	1.53	1.08–2.18	35/199	0.87	0.52–1.47	
67.8–3116	121/200	1.45	1.01–2.08	44/200	1.27	0.77–2.09	
Ln(propylparaben)		1.06	0.99–1.15		1.01	0.91–1.13	1.04 (0.92–1.16)
Butylparaben							
<LOD	185/288	1.00	Reference	60/288	1.00	Reference	
LOD–2.49	82/155	0.95	0.67–1.34	30/155	1.09	0.65–1.81	
2.50–173	75/155	0.78	0.55–1.12	33/155	1.14	0.69–1.88	
Ln(butylparaben)		0.92	0.85–0.99		1.01	0.90–1.13	0.91 (0.81–1.03)
Multiple parabens^c^		1.07	0.86–1.33		1.10	(0.81–1.50	0.97 (0.69–1.36)
*RASSF1A*							
Methylparaben							
1.04–73.3	19/199	1.00	Reference	137/199	1.00	Reference	
73.4–267	22/199	1.22	0.60–2.45	131/199	1.09	0.79–1.53	
270–3174	23/200	1.36	0.68–2.73	133/200	1.12	0.80–1.57	
Ln(methylparaben)		1.08	0.89–1.30		1.06	0.96–1.16	1.01 (0.83–1.22)
Propylparaben							
<LOD–12.4	16/199	1.00	Reference	126/199	1.00	Reference	
12.5–67.7	22/199	1.52	0.73–3.16	136/199	1.30	0.93–1.81	
67.8–3116	26/200	2.00	0.98–4.08	139/200	1.31	0.94–1.84	
Ln(propylparaben)		1.14	0.97–1.34		1.04	0.97–1.12	1.09 (0.93–1.28)
Butylparaben							
<LOD	38/288	1.00	Reference	207/288	1.00	Reference	
LOD–2.49	12/155	0.67	0.33–1.35	100/155	1.04	0.75–1.44	
2.50–173	14/155	0.71	0.35–1.44	94/155	0.89	0.64–1.25	
Ln(butylparaben)		0.93	0.79–1.09		0.94	0.87–1.01	0.99 (0.84–1.17)
Multiple parabens^c^		1.14	0.73–1.79		1.07	0.87–1.32	1.03 (0.65–1.64)
*TWIST1*							
Methylparaben							
1.04–73.3	132/199	1.00	Reference	24/199	1.00	Reference	
73.4–267	128/199	1.11	0.80–1.55	25/199	1.12	0.59–2.12	
270–3174	128/200	1.13	0.81–1.58	28/200	1.24	0.66–2.32	
Ln(methylparaben)		1.07	0.97–1.17		1.03	0.87–1.21	1.07 (0.89–1.27)
Propylparaben							
<LOD–12.4	116/199	1.00	Reference	26/199	1.00	Reference	
12.5–67.7	135/199	1.37	0.98–1.92	23/199	1.07	0.57–2.02	
67.8–3116	137/200	1.43	1.01–2.01	28/200	1.20	0.65–2.24	
Ln(propylparaben)		1.06	0.98–1.14		1.01	0.89–1.15	1.06 (0.93–1.22)
Butylparaben							
< LOD	200/288	1.00	Reference	45/288	1.00	Reference	
LOD–2.49	96/155	1.02	0.73–1.43	16/155	0.77	0.41–1.45	
2.50–173	92/155	0.92	0.66–1.28	16/155	0.63	0.33–1.22	
Ln(butylparaben)		0.95	0.88–1.03		0.88	0.76–1.02	1.10 (0.95–1.28)
Multiple parabens^c^		1.10	0.89–1.36		0.96	0.65–1.42	1.23 (0.81–1.87)

*Note*: LIBCSP population‐based women without breast cancer were frequency matched by age to women diagnosed with breast cancer between August 1, 1996 and July 31, 1997.

Abbreviations: APC, adenomatous polyposis coli; Ca, cases; Co, controls; LIBCSP, Long Island Breast Cancer Study Project; LOD, limit of detection.

^a^ Multinomial logistic regression ORs adjusted for age (continuous) and education (<high school/high school graduate, college, postcollege), menopausal status (premenopausal, postmenopausal), age at menarche (≤12, >12 years), parity and lactation history (nulliparous, parous/never lactated, parous/ever lactated), family history of breast cancer (none or at least one first degree relative), hormone replacement therapy use (ever, never), body mass index in the year before diagnosis (≤25.0, >25.0 kg/m^2^), and lifetime alcohol intake (nondrinkers, <15, 15–29, ≥30g*/*day).

^b^ Ratio of the odds ratio (ROR) indicating outcome heterogeneity from logistic regression models estimating the odds of hypomethylated tumor promoter status to hypermethylated promoter status among cases. In the absence of outcome heterogeneity, ROR = 1.

^c^ Multiple paraben analysis estimated using quantile g‐computation.

For BPB, the highest (vs. lowest) quantile was associated with a decreased odds of hypermethylated *CCND2* promoter‐defined BC (OR = 0.48, 95% CI = 0.25–0.93; CLR = 3.72) versus controls, and with an OR of hypomethylated *CCND2* promoter‐defined BC close to one (OR = 0.98, 95% CI = 0.70–1.37; CLR = 1.96). A one‐ln unit increase in BPB was associated with an OR of 0.79 (95% CI = 0.68–0.92; CLR = 1.35) for hypermethylated *CCND2* promoter‐defined BC and with an OR of 0.97 (95% CI = 0.90–1.05; CLR = 1.17) for hypomethylated *CCND2* promoter‐defined BC. There was a 55% increase in the odds of hypomethylated (vs. hypermethylated) *CCND2* promoter‐defined BC (ROR = 1.55, 95% CI = 1.04–2.32; CLR = 2.23) for a one ln‐unit increase in the multiple parabens.

We also observed elevated odds of hypomethylated tumor promoter‐defined BC for the highest (vs. lowest) tertiles PPB for most genes; however, the RORs were not statistically significant.

## 4 DISCUSSION

We examined whether global DNA methylation modified associations between urinary parabens and BC and examined whether parabens were associated with BCs having gene‐specific promoter methylation. We observed elevated odds of BC for MPB and BPB that were more pronounced among women with global DNA hypomethylation than among women with global DNA hypermethylation. We also observed elevated odds of BC among women with global DNA hypomethylation when we examined all parabens together; however, the estimate for the multiple parabens was attenuated as compared to the results for MPB and PPB individually, suggesting that the parabens may have sub‐additive effects (the weights for all three parabens were positive but unequal in the quantile g‐computation analysis). In our gene‐specific promoter methylation analysis, we observed elevated odds of BC for hypomethylated *CCND2* promoter methylation for MPB (highest vs. lowest quantiles) and lower odds for hypermethylated *CCND2* promoter methylation‐defined BC for BPB (highest vs. lowest quantile). PPB (highest vs. lowest tertile) was also associated with 40%–100% increases in the odds of BC with hypomethylated promoter methylation for most genes examined.

Our observations of increased odds of BC among women with global DNA hypomethylation are consistent with the currently proposed hypotheses, suggesting that global DNA hypomethylation increases genomic and chromosomal instability.^19^ Our results suggest that the carcinogenic effects of parabens may be exacerbated in the presence of genomic or chromosomal instability. In the gene‐specific tumor promoter methylation analysis, higher levels of MPB and PPB, and the multiple parabens were associated with higher odds of hypomethylated *CCND2* promoter‐defined BC compared to controls. *CCND2* is an oncogene that encodes cyclin D2, which helps to regulate the cell cycle G1/S transition.^39^ Hypomethylation of *CCND2* could potentially increase expression of *CCND2*,^20^ resulting in an increased risk of BC, and thus our results reported here are biologically plausible. Future studies, however, should confirm our findings and test hypotheses elucidating these mechanisms to further understand the role of DNA methylation in the associations between parabens and BC risk.

This study has a number of strengths, including the analysis of both global and gene‐specific tumor DNA methylation in a well‐characterized population of women with and without BC, and the use of an objective measure of paraben exposure. Our sample size was also relatively large, though we were likely underpowered to detect statistically significant interactions. To our knowledge, this is the first study to investigate the modifying effect of global DNA methylation on the associations between parabens and BC and the first to consider parabens in association with tumor promoter methylation. However, our results should be interpreted in light of limitations. First, the LIBCSP was a case‐control study and blood and urine samples were collected from women with BC after diagnosis but before the initiation of chemotherapy for most women. It is unclear whether paraben levels measured in urine samples collected close to diagnosis reflect the etiologically relevant period for breast carcinogenesis, which is likely to be years before diagnosis.^40^ Similarly, global methylation status may reflect shorter‐term temporal variation in epigenetic modification as a result of BC. However, in a previous analysis of the LIBCSP,^16^ women in the highest versus lowest quintile of LUMA methylation were found to have a twofold increased risk of BC, but LINE‐1 methylation, for which we observed stronger associations in our stratified analyses, was not associated with BC risk. Also, paraben exposure could potentially influence global DNA methylation status; however, a prospective design will be needed to disentangle the directions of association between parabens, global DNA methylation, and BC. Second, a single measure of paraben levels in urine samples may not be sufficient to adequately capture levels of chronic paraben exposure; however, parabens show fair to good intraclass correlations over weeks or months ranging from 0.42 to 0.61 for MPB and 0.32 to 0.55 for PPB.^41^, ^42^, ^43^ Third, LUMA and LINE‐1 are older measures of global DNA methylation. While both assays show a high correlation with the high‐performance liquid chromatography (HPLC)‐based gold standard and have the advantage of requiring far less DNA than HPLC,^17^ the biological differences between LINE‐1 and LUMA combined with technical differences in the assays could have introduced bias into the estimates of global DNA methylation.^18^ For example, LUMA relies on CpGs located within “CCGG” regions, which account for ∼8% of the human genome, whereas long interspersed elements account for ∼17%–20% of the human genome. This may result in a lower sensitivity of LUMA compared to LINE‐1. Additionally, neither assay provides information about where in the genome the methylated cytosines are located precluding our ability to comment on the importance of loci‐specific methylation status in these associations. Future studies should consider evaluating epigenome‐wide associations and interrogate BC‐associated loci that may modify paraben‐breast cancer associations. Last, the large number of statistical tests could have resulted in spurious associations; however, as we examined environmental agents known to exert physiologic effects, real associations are to be expected, and therefore, adjustments for multiple comparisons are unwarranted.^44^ Nonetheless, our results provide potential avenues for further exploration in future studies.

## 5 CONCLUSIONS

Herein, we examined the modifying effects of global DNA methylation on associations between urinary parabens and BC and examined associations between parabens and tumor promoter methylation‐defined BCs. Our results suggest parabens may be more potent carcinogens among women with global DNA hypomethylation, and that parabens may increase the risk of hypomethylated *CCND2* promoter methylation‐defined BCs. In 2006, the FDA reported the use of parabens in over 22,000 formulations.^45^ Consequently, the findings of this study are relevant as they have implications on the regulation of the use of parabens and other endocrine disrupting chemicals used in personal care and consumer products frequently used by women and may suggest that public health interventions are needed to reduce exposure to parabens and potentially the risk of BC.

## AUTHOR CONTRIBUTIONS

The study was conceptualized and designed by Humberto Parada Jr. Regina M. Santella, Alfred I. Neugut, and Susan L. Teitelbaum administered the Long Island Breast Cancer Study Project and acquired data. Humberto Parada Jr and Leili Sahrai analyzed the data. Humberto Parada Jr wrote the original draft. Leili Sahrai, Mary S. Wolff, Regina M. Santella, Jia Chen, Alfred I. Neugut, and Susan L. Teitelbaum reviewed and interpreted the results and provided editing. All authors read and approved the final manuscript.

## CONFLICT OF INTEREST

Dr. Neugut has consulted for Otsuka, GlaxoSmithKline, Eisai, Hospira, and United Biosource Corp., and has grant support from Otsuka. He serves on the medical advisory board of EHE Intl. The remaining authors declare no conflict of interest.

## Data Availability

The data that support the findings of this study are available from the corresponding author upon reasonable request.
